# Deep learning for diagnostic and clinical decision support in companion animals: a systematic review of CNN- and LSTM-based approaches

**DOI:** 10.3389/fvets.2026.1952589

**Published:** 2026-10-22

**Authors:** Juan J. Guevara-Flores, Jhonatan López-Rojas, José A. Vásquez–Coronel

**Affiliations:** 1 Facultad de Ingeniería en Sistemas e Informática, Universidad Tecnológica del Perú, Lima, Peru; 2 Centro de Apoyo Logístico al Investigador (CALI), Universidad Tecnológica del Perú, Lima, Peru

**Keywords:** artificial intelligence, computational infrastructure, deep learning, diagnostic imaging, disease detection, veterinary oncology

## Abstract

Early disease detection prior to the onset of advanced clinical signs remains a critical imperative in veterinary medicine. This systematic review provides a methodological synthesis of Convolutional Neural Networks and Long Short-Term Memory networks applied to diagnostic and clinical decision support in canine and feline patients, evaluating both algorithmic performance and clinical feasibility. Adhering to the PICO-CS framework and PRISMA 2020 guidelines, we queried Scopus and Web of Science for peer-reviewed studies published between 2021 and 2026, retrieving a final corpus of 57 primary studies. Eleven clinical domains were identified, with evidence heavily concentrated in Oncology (28.1%), Cardiology (15.8%), and Pulmonology (12.3%). CNN architectures predominated in spatial imaging modalities—including radiographs, clinical photography, ultrasonography, and microscopic imagery—whereas LSTM networks were deployed for temporal signals and sensor-derived telemetry. Binary classification tasks achieved the highest performance metrics (accuracy reaching up to 99%), whereas multi-class formulations exhibited broader variability due to dataset imbalance. Cross-study benchmarking revealed substantial heterogeneity in datasets, evaluation metrics, and validation protocols, while reliance on high-performance computational infrastructure poses a barrier to point-of-care deployment in resource-constrained clinics. We conclude that while deep learning architectures hold significant promise as clinical decision-support tools, transitioning to routine veterinary practice requires prospective external validation, representative open-access datasets, and lightweight models optimized for conventional clinical hardware.

## 1 Introduction

Contemporary veterinary medicine faces a critical challenge: its diagnostic paradigm remains predominantly reactive. Clinical interventions typically initiate only after overt symptoms manifest or through time-consuming laboratory procedures, such as complex genetic and molecular assays (1). This diagnostic lag adversely impacts patient prognosis and restricts early intervention in critical pathologies. To address these limitations, Machine Learning (ML) and Deep Learning (DL) offer viable frameworks for rapid disease prediction and diagnosis from large-scale datasets (2, 3). However, translating these theoretical algorithms into routine clinical practice remains a central challenge.

The motivation to apply these advanced architectures within veterinary medicine is heavily driven by the profound success of AI-based solutions across diverse general applications and human healthcare. In non-medical domains, for instance, fractionally optimized and attention-enhanced Convolutional Neural Networks (CNNs) have been successfully deployed for complex multi-output tasks, such as latent fingerprint classification (4), automated fruit and vegetable categorization (5), and the robust identification of nonlinear industrial systems like heat exchangers (6). More critically, in human medicine, tailored deep learning networks and transfer learning strategies have achieved unprecedented accuracy in diagnosing non-small cell lung cancer (7), chronic kidney disease (8), monkeypox (9), and malaria (10). Furthermore, explainable and spatially focused CNNs are revolutionizing the early, interpretable diagnosis of complex neurodegenerative conditions such as Alzheimer's disease (11, 12). The proven efficacy, adaptability, and interpretability of these algorithms in solving both general classification tasks and critical human health challenges serve as a compelling catalyst to explore their translational potential in veterinary diagnostics.

Predictive models have already demonstrated substantial utility in evaluating animal health across diverse data modalities. Advanced systems analyze biological fluids in felines (13), while ensemble learning frameworks predict canine endocrine disorders using routine laboratory metrics (14). Similarly, integrated point-of-care platforms evaluate peripheral blood smears with high precision (15). In diagnostic imaging, CNNs have achieved high performance in identifying cardiac abnormalities in dogs and cats (16, 17), while also being applied in Computed Tomography (CT) for nasal pathologies (18), ocular ultrasonography for cataract classification (19), and radiography for hip dysplasia assessment (20). In oncology, artificial intelligence has shown high efficacy in detecting mammary tumors (21), classifying lymphomas via microscopic imaging (22), and predicting mutational states from cell morphology (23). Additional applications encompass motion analysis for lameness detection (24) and automated recognition of dermatological conditions (25).

Despite recent progress, research on artificial intelligence in veterinary medicine remains highly fragmented. Comprehensive overviews provide broad perspectives on the field (26), alongside specialized studies focused on specific conditions, such as viral infections (2, 3). However, existing literature exhibits substantial heterogeneity in datasets, evaluation metrics, and experimental protocols, which hampers direct benchmarking and obscures the clinical feasibility of deploying specific models in small animal practice. Furthermore, it limits experimental reproducibility, as underlying datasets and methodological workflows are frequently inaccessible or inconsistent. Consequently, many proposed methodologies remain confined to experimental validation and have yet to transition into clinical workflows.

Given the absence of standardized evaluation criteria, this review systematically assesses deep learning methodologies applied to diagnostic and clinical decision support in dogs and cats. The scope focuses on CNNs and Long Short-Term Memory networks (LSTMs) for the analysis of spatial data, particularly medical images, and temporal data, including time-series records, in veterinary applications. Restricting the review to these two architectures provides a consistent basis for comparing reported model performance, clinical applications, and computational requirements. The thematic scope includes diagnostic imaging modalities such as radiographs, ultrasonography, CT, Magnetic Resonance Imaging (MRI), and microscopic histopathology. Non-imaging data, including Electroencephalography (EEG), wearable sensors, and genomic data, are also included when integrated into CNN or LSTM frameworks for diagnostic or clinical decision-support tasks. In these applications, temporal signals can be transformed into two-dimensional representations, such as spectrograms, and processed by CNNs for feature extraction. The review assesses reported model performance across clinical domains, including oncology, cardiology, and pulmonology, and examines the technological constraints associated with point-of-care implementation.

## 2 Methodology

To establish the methodological foundation of this systematic review and align with the PRISMA 2020 guidelines (27), the eligibility criteria were structured around a PICO-CS (Population, Intervention, Comparison, Outcome, Context, and Study Design) framework. No prospective review protocol was registered in a public registry for this systematic review. However, the search strategy, eligibility criteria, research questions, and analytical framework were prospectively established prior to executing the literature search and data extraction procedures. The Population comprised companion animal pathologies, with a specific focus on canine and feline subjects. The intervention included deep learning architectures, specifically CNNs and LSTM networks. The outcome encompassed diagnostic and clinical decision-support capabilities. The comparison component was not incorporated as a database search concept because the review focused on characterizing the clinical utility of these neural networks rather than evaluating their direct diagnostic equivalence or superiority relative to conventional approaches. Including comparison-related Boolean terms could have reduced search sensitivity and limited retrieval of eligible primary studies. Nevertheless, Section 3 presents qualitative comparisons with conventional clinical procedures and diagnostic standards, including histopathological confirmation, reference standards, and board-certified specialist consensus. Study design was an explicit eligibility criterion and was restricted to peer-reviewed primary empirical studies. These included retrospective and prospective diagnostic accuracy studies, cohort studies, and clinical trials. Narrative reviews, theoretical models, and single-animal case reports were excluded. Finally, Context was incorporated as an additional dimension to characterize the operational, computational, and clinical environments associated with model development or deployment, including hardware availability, computing resources, and clinical workflow integration.

To address each component of this framework, we formulated the following research questions:

- Which pathologies or diseases are diagnosed in companion animals?
- How are CNN and LSTM neural networks applied for diagnostic and clinical decision support in companion animals?
- What metrics and outcomes are obtained when applying these models to companion animal pathologies?
- In what clinical or technological contexts are CNN and LSTM models deployed for pet disease diagnosis?

These queries were formulated independently to analyze each dimension of the problem from a specific perspective before synthesizing them into a comprehensive framework. The examination of specific diseases delineated the clinical scope of the review, focusing the investigation on pathologies supported by robust scientific evidence. Similarly, the study of neural networks focused exclusively on understanding the technical implementation of CNN and LSTM architectures within the veterinary domain. Evaluating the reported performance metrics facilitated a quantitative assessment of algorithmic accuracy and practical utility, while analyzing the clinical setting elucidated operational constraints and integration feasibility in current veterinary practice. Synthesizing these factors led to our central research question: How are CNN and LSTM neural networks applied for diagnostic and clinical decision support in companion animal pathologies within clinical veterinary settings?

### 2.1 Information sources and search strategy

A systematic literature search was conducted across the Scopus and Web of Science (specifically, the Web of Science Core Collection) databases. These platforms were selected due to their internationally recognized indexing standards and high-impact, peer-reviewed scientific coverage (28). This was critical for our study, which required high technical rigor in both computer science and veterinary medicine. The final search across both databases was executed on April 23, 2026.

To retrieve relevant literature, a search strategy targeting article titles, abstracts, and keywords was constructed based on the PICO-CS components. The core conceptual query was: (Disease OR Pathology) AND (CNN OR “Convolutional Neural Network” OR LSTM OR “Long Short-Term Memory”) AND (Prediction OR Detection OR Classification OR Diagnosis) AND (Dog OR Canine OR Cat OR Feline OR Veterinary). This query was formulated exclusively using free-text keywords without the application of controlled vocabularies, ensuring broad retrieval across heterogeneous AI nomenclature. Additionally, neither citation chaining nor manual searching of reference lists was performed. The conceptual query was subsequently translated into database-specific syntax (using TITLE-ABS-KEY for Scopus and TS for Web of Science). To ensure full methodological reproducibility, the exact search strings applied for each database are provided in the Appendix.

Four primary filtering criteria were applied during the retrieval process. First, document types were restricted exclusively to peer-reviewed journal articles, excluding conference proceedings and book chapters to ensure analytical depth. Second, the publication window was restricted to the last five years (2021–2026) to reflect recent advancements in deep learning architectures. Third, only articles published in English were considered, as English remains the primary language for international scientific communication in artificial intelligence and veterinary research. The initial database search included Open Access filters to facilitate immediate full-text evaluation. Following title and abstract screening, full-text retrieval for each retained record was systematically conducted using reference management software (Zotero) via DOI identifiers. When full-text files were unavailable through initial indexing, Google Scholar and ResearchGate were consulted as secondary repositories. This multi-tiered retrieval procedure was implemented to maximize full-text retrieval and minimize potential availability bias. Studies were excluded only when the full-text report could not be retrieved from any of these sources after exhaustive attempts.

### 2.2 Data management and selection criteria

Initial data management and deduplication were conducted using Microsoft Excel matrices. Raw query exports were loaded, duplicate entries were identified and removed, and initial screening was performed to assess topic relevance. Full-text availability was then verified prior to in-depth eligibility assessment based on predefined selection criteria. Zotero was utilized as the primary reference manager for library organization, full-text PDF review, and citation management.

To ensure conceptual clarity during study selection and synthesis, strict operational definitions were established for the tasks performed by the evaluated models. Detection was defined as the algorithmic identification and spatial localization of an anomaly or region of interest within a clinical input (e.g., bounding boxes around a tumor). Classification referred to the categorization of an entire input instance or previously isolated features into predefined pathological classes (e.g., benign vs. malignant). Prediction was operationalized as the forecasting of a clinical state based on temporal or sequential data patterns prior to the definitive onset of clinical signs (e.g., anticipating a seizure). Diagnosis was considered the overarching clinical objective, acting as a composite of detection, classification, and prediction when integrated into a decision-support framework by a veterinary professional.

To guide study selection, specific inclusion criteria (IC) and exclusion criteria (EC) were established. We included only studies focused on dogs and cats, excluding livestock, production animals, and wildlife species. Included works were required to employ deep learning architectures—specifically CNNs and LSTMs—for diagnostic and clinical decision support, ensuring relevance to early clinical detection. Conversely, studies were excluded if they failed to address the central research question. Criterion EC1 excluded studies focusing on survival prediction or treatment efficacy, as these address patient prognosis rather than initial diagnosis. Criterion EC2 removed works based on traditional machine learning or non-relevant algorithms retrieved due to nomenclature overlap (e.g., Cat Swarm Optimization), ensuring a strict focus on deep neural networks. Criterion EC3 excluded qualitative studies and literature reviews, ensuring that all synthesized primary studies presented empirical data and quantitative metrics.

### 2.3 Methodological quality and risk of bias assessment

We assessed methodological risk of bias across the 57 included primary studies using a six-criterion framework adapted from the principles of PROBAST + AI (Prediction model Risk Of Bias Assessment Tool for Artificial Intelligence) (29). PROBAST + AI evaluates potential sources of bias in prediction studies by assessing participants, predictors, outcomes, and analysis. The present framework adapted these principles to the characteristics of artificial intelligence studies in companion animal diagnosis. TRIPOD + AI (30) was used separately as a reporting guideline to assess the completeness of information required to understand model development and evaluation. Thus, TRIPOD+AI was not used to determine the risk-of-bias scores, and reporting completeness was assessed separately from methodological risk of bias.

We assessed each study using six predefined criteria, designated Q1 to Q6. Each criterion received a score from 0 to 2, with 2 indicating low risk of bias, 1 indicating moderate risk, and 0 indicating high risk. Q1 assessed participant selection and population representation. A score of 2 corresponded to a well-defined clinical population with appropriate eligibility criteria and adequate representation of the target population; a score of 1 corresponded to a clearly defined population from a single center or with limited clinical variability; and a score of 0 corresponded to a poorly characterized or clearly unrepresentative population. Q2 assessed predictor definition and model specification. A score of 2 corresponded to clearly defined predictors or input variables that were appropriately measured and specified for the prediction task; a score of 1 corresponded to partially defined predictors or limited information on their measurement; and a score of 0 corresponded to poorly defined or inadequately described predictors or input variables.

Q3 assessed the validation strategy. A score of 2 corresponded to model performance evaluated on an independent external dataset; a score of 1 corresponded to validation based on internal cross-validation or a hold-out test set; and a score of 0 corresponded to performance reported only on the data used for model development without an independent validation procedure. Q4 assessed whether the reported performance measures matched the prediction task. A score of 2 corresponded to the use of appropriate task-specific performance measures that adequately addressed the main aspects of model performance. For classification, appropriate measures included F1-score, Matthews correlation coefficient, area under the receiver operating characteristic curve, sensitivity, specificity, or other metrics suited to the prediction task. For object detection, mean Average Precision was considered appropriate, whereas Dice Similarity Coefficient or Intersection over Union was considered appropriate for segmentation. For regression and temporal prediction, Mean Absolute Error or Root Mean Squared Error was considered appropriate. A score of 1 indicated that the study reported task-specific metrics but did not adequately address important aspects of performance, whereas a score of 0 indicated that the reported metrics were inadequate for the prediction task. We interpreted the specific performance measures according to the characteristics of each prediction task rather than applying a single metric across all tasks.

Q5 assessed the potential for data leakage. A score of 2 corresponded to data partitioning at the patient or animal level, preventing observations from the same subject from appearing across the training and test sets; a score of 1 corresponded to an insufficiently described partitioning procedure in which potential leakage could not be excluded; and a score of 0 corresponded to a partitioning strategy that allowed observations from the same subject to appear across the training and test sets, creating a potential source of information leakage. Q6 assessed clinical applicability. A score of 2 corresponded to model evaluation using a clinically defined population and conditions relevant to the intended diagnostic use; a score of 1 corresponded to a discussion of clinical applicability without assessment using study data; and a score of 0 corresponded to the absence of information addressing the clinical use of the model. We applied the same scoring principles across classification, detection, segmentation, regression, and temporal prediction studies, while interpreting each criterion according to the characteristics of the specific prediction task. The signaling questions used to guide the assessment of the six criteria are presented in Table 1.

**Table 1:** Signaling questions used for the methodological risk-of-bias assessment.

Criterion	Signaling question
Q1	Did the study include a well-defined clinical population with appropriate eligibility criteria and adequate representation of the target population?
Q2	Were the predictors or input variables clearly defined, appropriately measured, and adequately specified for the prediction task?
Q3	Was model performance evaluated using a validation strategy appropriate for assessing performance beyond the data used for model development?
Q4	Were the reported performance measures appropriate for the specific prediction task and sufficient to characterize its main aspects of model performance?
Q5	Did the data partitioning strategy prevent observations from the same patient or animal from appearing across the training and test sets?
Q6	Was the model evaluated under conditions and in a population relevant to its intended clinical diagnostic use?

We summed the six criterion scores to obtain a total score ranging from 0 to 12. Scores from 9 to 12 indicated low methodological risk of bias, scores from 5 to 8 indicated moderate risk, and scores from 0 to 4 indicated high risk. Two authors independently assessed the included studies and resolved disagreements through discussion with the third author. Table 8 in the Appendix section presents the complete study-level application of these criteria across the 57 included studies.

### 2.4 Screening and data extraction procedures

To reduce the potential for selection bias, a blinded and independent screening process was conducted by the three authors. The three authors independently screened the titles and abstracts of all retrieved records to assess their initial relevance. Studies that met the initial screening criteria were subsequently evaluated at the full-text stage by the same authors, working independently and without access to each other's assessments. Each author classified the articles in Microsoft Excel according to the predefined inclusion and exclusion criteria. Disagreements arising during the full-text assessment were resolved through discussion and consensus among the three authors. Following the final selection, the three authors collaboratively performed the data extraction and jointly verified the methodological and technical information reported in the included studies.

The reporting of the selection process adhered to the PRISMA 2020 (Preferred Reporting Items for Systematic Reviews and Meta-Analyses) guidelines to ensure methodological transparency (27, 31), as illustrated in Figure 1. The search initially identified 225 records (153 from Scopus and 72 from Web of Science). After removing 67 duplicate records via reference management tools, 158 unique records remained. Title and abstract screening led to the exclusion of 93 studies that did not align with the research scope (e.g., human medicine, livestock applications, or unrelated optimization algorithms), leaving 65 articles eligible for retrieval. Of these, 1 full-text document could not be retrieved, leaving 64 reports for full-text eligibility assessment. Following rigorous full-text review, 7 articles were excluded: 2 under EC1, 4 under EC2, and 1 under EC3. Ultimately, 57 primary studies met all criteria and were included in this systematic review for data extraction and synthesis.

**Figure 1:**
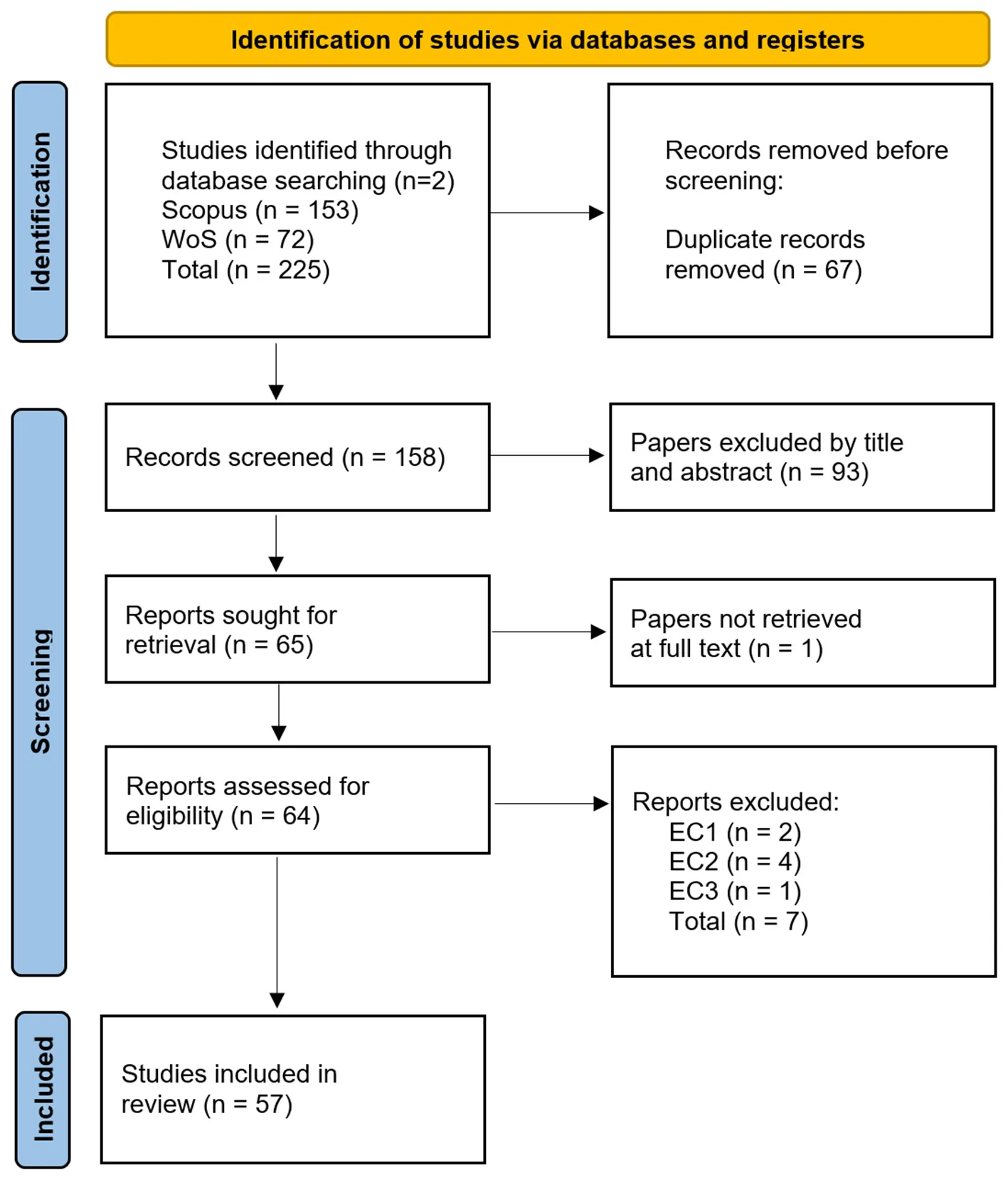
PRISMA flow diagram outlining the study selection process.

Despite the methodological rigor applied, certain scope limitations are acknowledged. Excluding non-English publications presents a potential constraint, though it ensured adherence to global technical standards. Restricting the search to Scopus and Web of Science carries the risk of omitting relevant studies indexed in other repositories. Excluding book chapters and conference papers prioritized peer-reviewed journal depth over secondary literature formats. Finally, restricting the search to Open Access publications from 2021 onwards ensured technological currency, though at the cost of excluding closed-access studies or earlier foundational models.

## 3 Results

From the 57 selected primary studies, key findings regarding the practical application of deep learning in veterinary diagnosis were extracted. The analysis was structured around four thematic axes: the clinical domains and pathologies most frequently addressed in the literature, the relationship between model architectural families and clinical data modalities, the performance ranges reported across specific computational tasks, and the technological and geographical contexts in which the research was conducted.

### 3.1 Clinical domains and pathologies investigated

Analysis of the selected publications identified 11 distinct clinical domains in which CNN and LSTM architectures have been applied for diagnostic and clinical decision support in companion animals. The data reveal a strong preference for specialties with digitized and highly standardized clinical records, resulting in a notable concentration across three primary areas: Oncology, Cardiology, and Pulmonology.

Within our review, Oncology encompassed the largest number of clinical pathologies. We identified multiple approaches for classifying mammary tumors, with some studies focusing on general frameworks (32–34) and others on specific analyses (35). Likewise, solutions have been developed for the histopathological differentiation of mast cell tumors (36–38) and lymphomas (22, 39). Osteosarcoma detection (40) and the prediction of mutational states from cell morphological features (41) are also addressed. This high concentration of oncological studies likely stems from the availability of robust histopathological datasets and the clinical motivation to automate tumor staging, which has traditionally suffered from inter-observer subjectivity.

In Cardiology, the strongest trend centers on automating radiographic indices—such as the Vertebral Heart Size (VHS) and Cardiac-to-Thoracic Ratio (CTR)—for early cardiomegaly detection. Various methodological approaches have evaluated these indices to enhance diagnostic accuracy (16, 42, 43), while other researchers validated them under specific clinical conditions (44, 45). This specialty also covers the classification of myxomatous mitral valve disease (MMVD) via phonocardiographic signals (17) and the screening of feline hypertrophic cardiomyopathy from thoracic radiographs (46–48). Notably, a prominent global pattern is the disparity between the species studied. Research has predominantly targeted canines, whereas felines are consistently represented only in Cardiology and Pulmonology. As shown in Figure 2, this asymmetrical distribution directly reflects data availability and feasibility.

**Figure 2:**
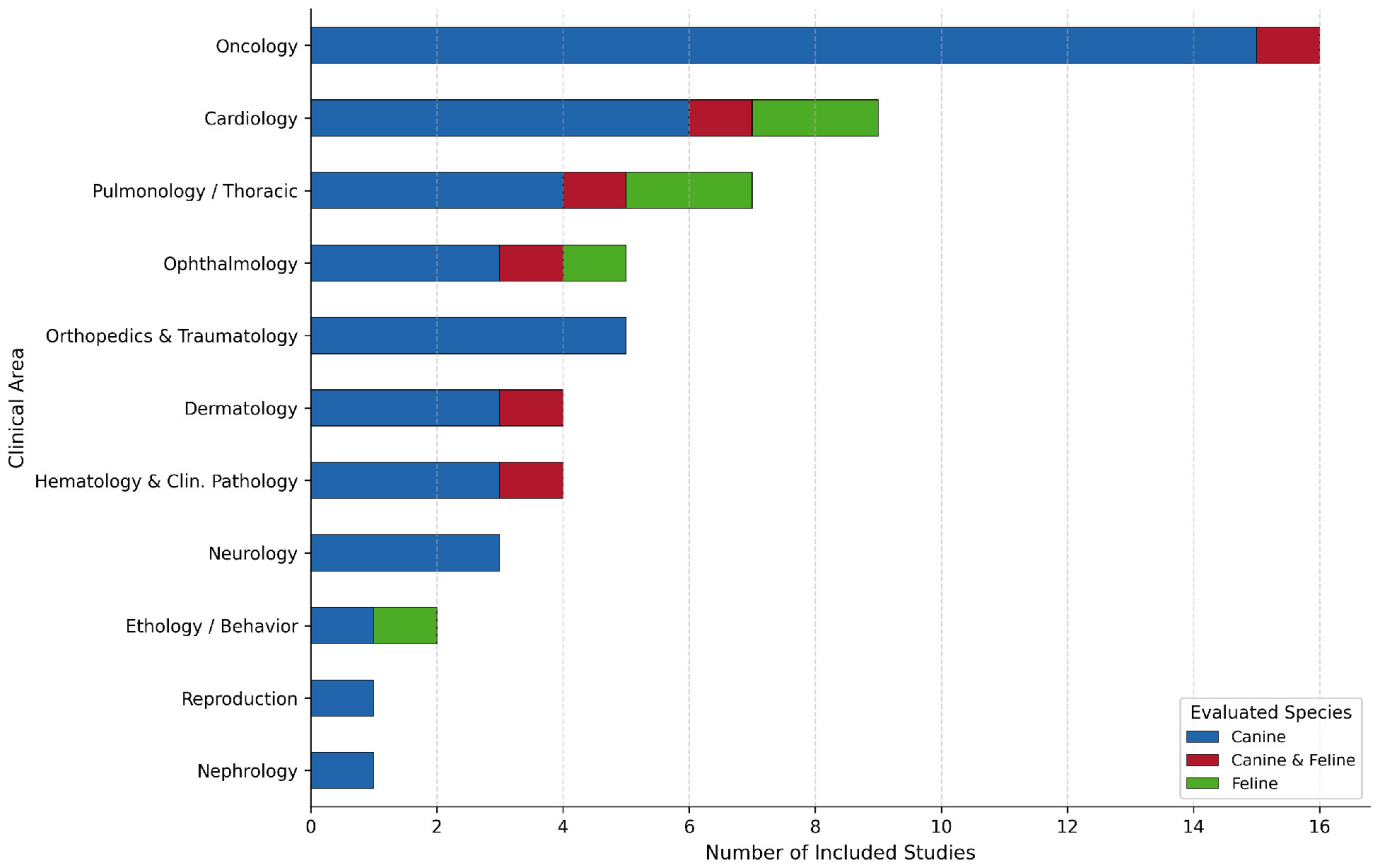
Distribution of clinical domains investigated using AI in companion animals, categorized by species evaluated.

Pulmonology groups research focused on detecting pleural effusion via ultrasonography (49), alongside multiple models for identifying diffuse pulmonary abnormalities on radiographs, ranging from pioneering work on alveolar patterns (50, 51) to more recent implementations on consolidations (52, 53). Additional applications include automated assessment of radiographic positioning (54) and the diagnosis of coccidioidomycosis (55).

More targeted applications further confirm the flexibility of these tools. In Ophthalmology, deep learning models have addressed complex pathologies where early diagnosis is critical to preserving vision. Automated diagnosis of retinal detachment (56) and severity classification of cataracts (19) have been explored. Studies aimed at detecting subtle retinal alterations (57–59) were also identified. These works demonstrate that AI can serve as a diagnostic aid during ophthalmological evaluation, particularly in procedures requiring specialized equipment and clinical expertise.

In Orthopedics, an expansion of investigated applications was observed. Beyond lesion detection, models were evaluated to identify and classify structural alterations, such as hip dysplasia (60) and elbow dysplasia (20). These tasks are particularly relevant because their interpretation exhibits inter-examiner variability. Proposals to analyze gait via video (61) and measure muscle wound volume (24, 62) were also found, which could facilitate rehabilitation monitoring.

In Clinical Pathology, computer vision was primarily applied to the automated analysis of blood smears. Models were utilized to evaluate erythrocyte morphology (63) and differentiate leukocyte types (15, 64), two tasks that typically require manual review. The detection of hemoparasites such as babesia (65) was also addressed, a relevant application in regions where vector-borne diseases are prevalent.

Applications were also identified in Dermatology, Neurology, and Ethology, although these domains had a smaller presence among the selected studies. In Dermatology, clinical photographs were used to classify skin lesions (66–68) and specific conditions such as pododermatitis (25). In Neurology, multimodal data were combined to study spinal cord pathologies (69, 70) and predict epileptic seizures from electroencephalograms (71). Meanwhile, ethological studies analyzed temporal signals from wearable sensors to assess activity, welfare, and separation anxiety (72, 73). Finally, applications in Nephrology (74) and Reproduction (75) were identified, expanding the range of clinical domains covered in this review.

Table 2 summarizes the identified clinical domains, investigated pathologies, included species, and input data types.

**Table 2:** Distribution of clinical domains investigated using CNN and LSTM architectures.

Clinical domain	Identified pathologies	Species	Input type	References
Oncology	Mammary tumors, mast cell tumors, lymphomas, osteosarcomas, skin tumors	Canine and feline	Genomic data, clinical photos, microscopic images, CT scans	(18, 22, 23, 32–41, 58, 76, 77)
Cardiology	Cardiomegaly, MMVD, hypertrophic cardiomyopathy	Canine and feline	Radiographs, audio signals	(16, 17, 19, 42–48, 50)
Pulmonology	Pleural effusion, pulmonary lesions, coccidioidomycosis	Canine and feline	Ultrasonography, radiographs	(49, 51–55)
Ophthalmology	Cataracts, retinal detachment, ocular surface lesions	Canine and feline	Ultrasonography, clinical photos	(56, 57, 59, 73)
Orthopedics	Hip/elbow dysplasia, lameness, muscle wounds, osteoarthritis	Canine	Clinical photos, radiographs, temporal signals	(20, 24, 52, 60–62)
Hematology	Erythrocyte morphology, leukocyte classification, babesiosis	Canine and feline	Microscopic images	(15, 63–65)
Dermatology	Bacterial/fungal dermatitis, pododermatitis, skin lesions	Canine and feline	Clinical photos	(25, 66–68, 78)
Neurology	Epilepsy, IVDD, spinal cord pathologies	Canine	Radiographs, MRI scans, temporal signals	(34, 69–71)
Ethology	Separation anxiety, activity monitoring	Canine and feline	Temporal signals	(69, 72)
Nephrology	Chronic kidney disease	Canine	Ultrasonography	(74)
Reproduction	Estrus cycle classification	Canine	Vaginoscopic images	(75)

Across the reviewed studies, dogs were more heavily represented than cats. This disparity may stem from a greater availability of clinical records and diagnostic imaging from canine patients for model training. Research was also concentrated in Oncology and Cardiology, likely associated with the availability of standardized clinical data and the demand to support complex diagnostic decision-making.

### 3.2 Architectures and clinical data modalities

An integrated analysis of clinical data modalities and network architectures revealed two primary trends. Across the selected studies, CNNs were predominantly deployed for spatial data processing, particularly clinical imaging, whereas LSTM and RNN architectures were concentrated on temporal signal analysis. This distribution demonstrates that architectural selection is intrinsically linked to the underlying data modality.

ResNet was the most frequently used architecture, with applications across six of the seven evaluated data modalities, particularly in radiographs and microscopic images. Its widespread adoption is attributable to residual connections, which enable the training of deep architectures while mitigating gradient degradation, thereby facilitating complex feature extraction from clinical images with high intra-class variability.

Architectural diversity is particularly pronounced in microscopic imaging, as illustrated in Figure 3. Alongside ResNet, YOLO and R-CNN frameworks were utilized for mitotic figure detection (37, 76) and specific cellular structure localization (15, 77). Notably, U-Net was employed for tumor region segmentation (39, 40), Inception for multi-class neoplasm classification (36), and Vision Transformers to capture long-range spatial dependencies in histopathological specimens (34, 58). Microscopic imaging yielded the broadest diversity of algorithmic architectures primarily because it encompasses classification, object detection, and semantic segmentation tasks simultaneously.

**Figure 3:**
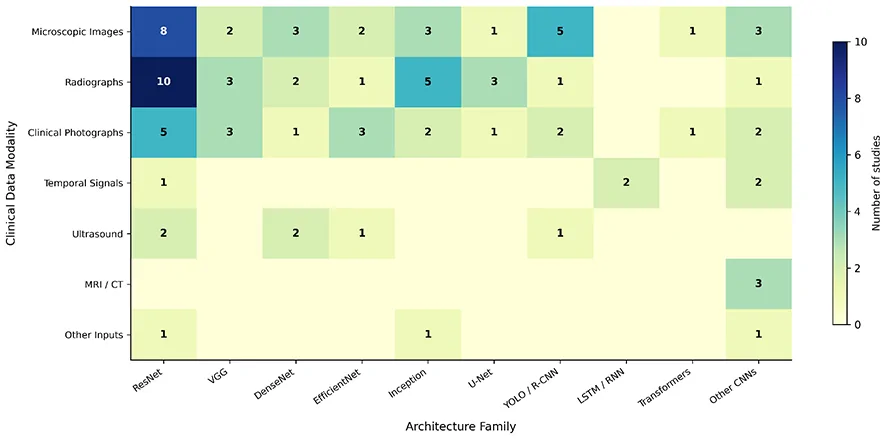
Relationship between clinical data modalities and architectural families.

In radiography, the architectural profile shifts toward a higher utilization of Inception and U-Net, applied respectively for thoracic pathology classification (43, 48, 51) and cardiac silhouette segmentation (44, 46). The absence of Transformers in this modality contrasts with their adoption in microscopic and clinical photography, which may be associated with the lower textural complexity of radiographic images.

Clinical photography shares a broad architectural spectrum with microscopic imaging but features a higher proportion of EfficientNet models (67, 68). Designed to optimize the trade-off between performance and computational efficiency, this architecture aligns well with mobile point-of-care image acquisition in veterinary practice.

The most substantive distinction between architectural families occurs in temporal signal processing. Across the reviewed literature, LSTM and RNN models were applied exclusively to sequential data. Applications included epileptic seizure prediction (71), behavioral monitoring via accelerometers (72, 73), murmur classification associated with mitral valve disease (17), and kinematic gait analysis (61). Hybrid frameworks were also identified, wherein temporal signals were transformed into spectrograms for subsequent 2D-CNN processing, thereby recasting signal patterns as image representations for computer vision pipelines.

Ultrasonography, MRI, and CT exhibited lower architectural diversity compared to other clinical modalities. Studies utilizing ultrasonography relied on DenseNet, EfficientNet, and YOLO for classification and specific structure detection (19, 49, 74). In MRI and CT, 3D-CNN architectures were predominantly applied for anatomical structure analysis and volumetric segmentation (18, 66, 70).

To categorize studies by dataset size, three descriptive tiers were defined: high (>1,000 cases or images), medium (500–1,000 cases or images), and low (< 500 cases or images). These thresholds serve as comparative benchmarks rather than universal sufficiency requirements for training neural networks. Applying this scheme revealed a bimodal concentration in the low and high ranges, with underrepresentation in the medium range, highlighting substantial disparities in dataset scale across veterinary AI research.

Detailed architectural distributions, diagnostic examples, and sample size tiers across modalities are summarized in Table 3.

**Table 3:** Technological analysis according to clinical data modality.

Data type	Architectures	Diagnostic examples	Sample range	References
Microscopic images	DenseNet, Inception, MobileNet, ResNet, VGG, YOLO	Lymphomas, mast cell tumors, babesiosis, erythrocyte morphology	High	(22, 32, 38, 63–65)
	Faster R-CNN, Inception, ResNet, EfficientNet	Neoplasms, mitosis detection, skin tumors	Medium	(36, 39, 77)
	CLAM, DenseNet, EfficientNet, MobileNet, ResNet, RetinaNet, U-Net, YOLO	Osteosarcoma, retinal degeneration, cellular analysis, necrosis	Low	(15, 23, 34, 35, 37, 40, 58, 76)
Radiographs	DenseNet, EfficientNet, Inception, MobileNet, ResNet, VGG	Thoracic pathologies, MMVD, cardiomegaly, coccidioidomycosis	High	(42, 44, 48, 50, 51, 53, 55, 60)
	Inception, Mask R-CNN, ResNet, U-Net	Atrial enlargement, hip dysplasia, pulmonary patterns	Medium	(16, 20, 43, 45, 46, 52)
	1D-CNN, InceptionResNet, MobileNet, ResNet, U-Net, Xception	IVDD, HCM, radiographic positioning	Low	(47, 54, 69)
Clinical photos	DenseNet, EfficientNet, Inception, ResNet, Swin-T, VGG, Xception, YOLO	Retinal detachment, gait abnormalities, ocular surface	High	(24, 33, 56, 57)
	EfficientNet, MobileNet, ResNet, YOLO	Pododermatitis, neoplasms, dermatitis	Medium	(25, 68, 78)
	CNN, EfficientNet, Inception, ResNet, U-Net, VGG	Wound status, skin lesions, multispectral dermatosis, ocular lesions	Low	(59, 62, 66, 67)
Temporal signals	1D-CNN, LSTM	Activity monitoring, separation anxiety	High	(72, 73)
	CNN, LSTM, ResNet, PaSST	Seizure prediction, mitral murmur, lameness	Low	(17, 61, 71)
Ultrasonography	AlexNet, DenseNet, EfficientNet, ResNet	Cataracts	High	(19)
	DenseNet, MobileNet, ResNet, YOLO	Emergency POCUS, kidney disease	Low	(49, 74)
MRI/CT	CNN, ResAttNet	Spinal cord pathologies, rhinitis, nasal neoplasia	High	(18, 70)
Other inputs	1D-CNN, Inception, ResNet, XGBoost	Estrus cycle, cross-species genomics	Low	(41, 75)

The selection of deep learning architectures depends almost exclusively on the nature of the clinical data modality. CNNs overwhelmingly predominate because veterinary medicine is fundamentally visual, relying heavily on radiographs, ultrasonography, and microscopy. The widespread success of robust backbones such as ResNet highlights that veterinary medical images exhibit substantial noise and intra-class variability, requiring deep residual architectures to preserve critical diagnostic features. Furthermore, the marked bimodal distribution in sample sizes underlines the lack of centralized public repositories, forcing researchers to rely on single-center hospital collections and hindering progress in less common pathologies.

### 3.3 Evaluation metrics and reported performance

Model performance varied substantially depending on the complexity of the computational task. Overall, simpler tasks, such as binary classification, achieved the highest performance peaks, whereas multi-class classification presented significant challenges due to class imbalance.

For binary classification problems, Accuracy emerged as the predominant metric (ranging from 0.82 to 0.99). The Area Under the ROC Curve (AUC) varied from 0.69 to 0.99, demonstrating notable variation in model fit across studies. Within binary classification literature, F1-scores ranged between 0.85 and 0.99, while sensitivity values spanned from 0.82 to 0.97.

The highest accuracy and AUC values were observed in studies utilizing thoracic radiographs for cardiomegaly detection (16, 42, 45) and those analyzing microscopic images for tumor classification (23, 32, 35). Conversely, lower AUC values were reported in investigations on pulmonary coccidioidomycosis (55) and ultrasonographic evaluation of chronic kidney disease (74). These discrepancies may relate to sample sizes, class distributions, or validation protocols. However, the synthesized evidence does not allow these variations to be attributed to a single definitive factor.

In multi-class classification tasks, accuracy ranged between 0.78 and 0.99, while F1-scores fell between 0.68 and 0.95–ranges generally lower than those observed in binary classification. Macro-F1 scores were also reported, with values between 0.68 and 0.94. In contrast, the Matthews Correlation Coefficient (MCC) saw limited adoption across the reviewed literature. Its sparse usage hinders robust model comparison when class distributions are highly skewed, particularly in histopathological differentiation (36, 38) and leukocyte classification (15, 64).

For object detection tasks, mean Average Precision (mAP) values ranged from 0.87 to 0.97, F1-scores spanned from 0.75 to 0.84, and sensitivity ranged between 0.91 and 0.97. These metrics were documented in applications such as mitotic figure identification (37, 76) and pododermatitis detection from clinical photographs (78). Figure 4 illustrates a comparison of the performance ranges categorized by computational task.

**Figure 4:**
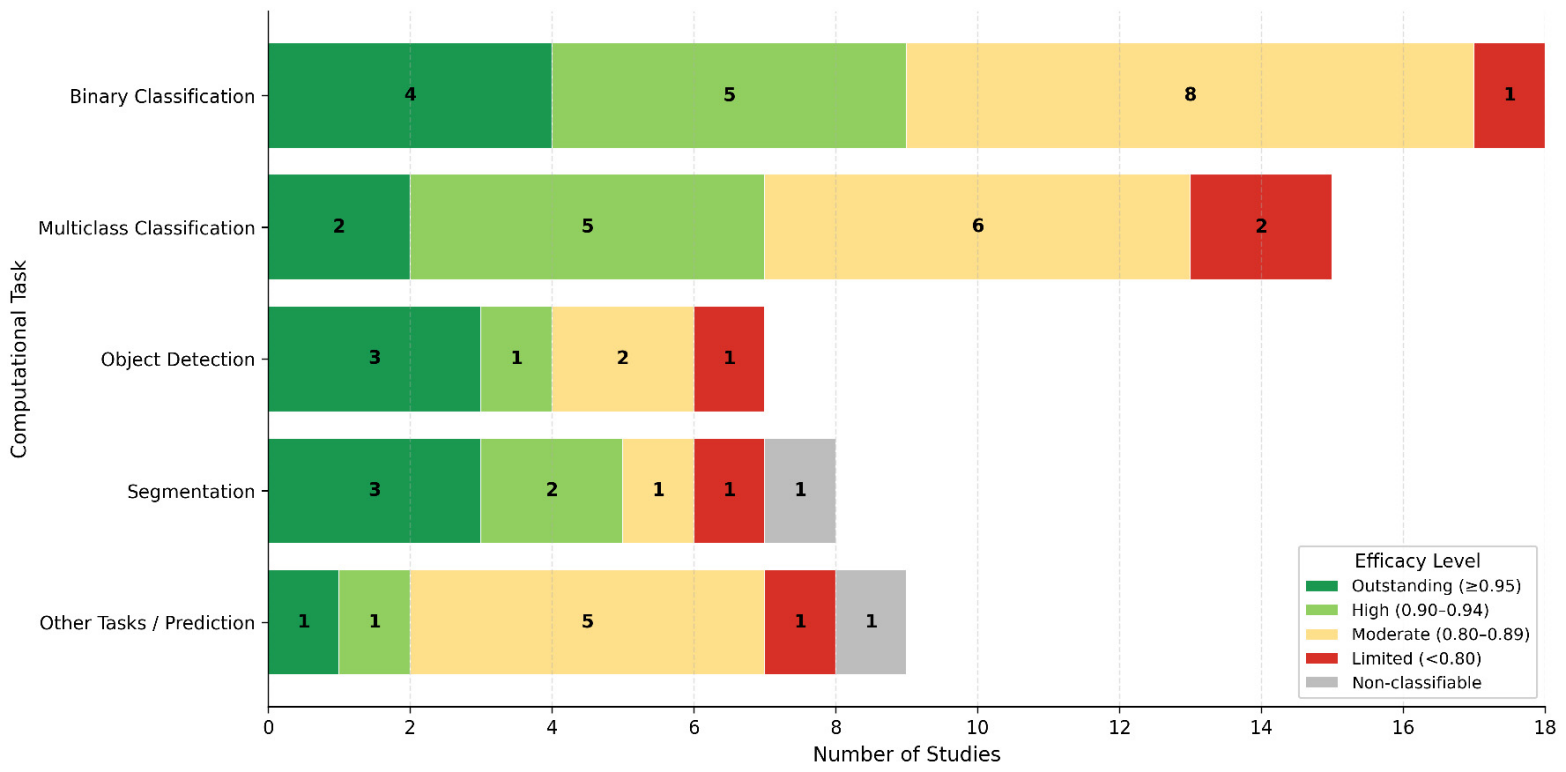
Distribution of performance levels by computational task.

In segmentation tasks, the most frequently reported metrics were the Dice Similarity Coefficient (Dice/DSC) and Intersection over Union (IoU). Dice/DSC scores ranged from 0.58 to 0.97, while IoU values spanned between 0.58 and 0.95. Lower performance bounds were observed in studies segmenting tumor regions in histopathological images (39, 40) and delineating the cardiac silhouette on thoracic radiographs (44, 46). These applications involve anatomical structures whose boundaries exhibit inter-observer variability, though not all studies evaluated this subjectivity uniformly.

Temporal prediction tasks exhibited greater metric diversity, constraining direct cross-study comparisons. In addition to accuracy and F1-score, sensitivity was reported with values between 0.62 and 1.00. This group encompassed heterogeneous applications, including epileptic seizure prediction (71), behavioral monitoring (72, 73), and genomic prediction (41). Because these tasks utilized distinct data modalities and clinical endpoints, their results must be interpreted independently. Furthermore, two studies reported regression metrics that are not directly comparable to classification tasks: one reported a Mean Absolute Percentage Error (MAPE) of 5% to 30% for wound volume quantification (62), while another reported a mean bias of −0.09 vertebrae in cardiac radiograph assessment (44).

A comprehensive breakdown of model performance, detailed by architectural family and corresponding evaluation metrics, is presented in Table 4.

**Table 4:** Detailed performance results by computational task and best-reported architecture.

Computational task	Primary architecture	Input type	Reported performance	References
Classification	DenseNet-121	Point-of-care ultrasound	Accuracy: 97.0%	(49)
	DenseNet-161	Whole slide images	Accuracy: 92.7%	(35)
	EfficientNet	Whole slide images	Accuracy: 85.0%	(32)
	EfficientNet B1–B4 ensemble	Elbow radiographs	Accuracy: 98.0%	(60)
	Ensemble-C	Thoracic radiographs	AUC: 0.909	(42)
	CLAM (MIL)	Whole slide images	MCA: 0.870/AUC: 0.940	(58)
	ResNet	Thoracic radiographs	AUC: 0.991	(53)
	ResNet-50v2	Ocular fundus photos	AUC: 0.919	(57)
	ResNet-50v2	Thoracic radiographs	Accuracy: 82.0%	(45)
	VGG-16	Thoracic radiographs	Accuracy: 88.7%	(55)
	VGG-16	Blood smears	Accuracy: 97.8%	(65)
	ViT	Fine needle aspirate cytology	AUC: 0.940	(22)
	Xception	Clinical photographs	Accuracy: 98.9%	(33)
	Xception	Thoracic radiographs	AUC: 0.917	(47)
	ResNet-50	Thoracic radiographs	AUC: 0.830	(16)
	1D-CNN	IMU sensor signals	Accuracy: 98.9%	(72)
	Stacked LSTM + Fuzzy-CEP	Accelerometer signals	F1-Score: 0.860	(69)
	Swin-T	Clinical photographs	Accuracy: 84.1%	(56)
	1D-CNN (cTULIP)	RNA-seq genomic profiles	F1-Score: 0.889	(41)
	PaSST/ResNet38	Audio signals	Accuracy: 94.1%	(17)
	DenseNet-121	Blood smears	F1-Score: 0.920	(64)
	DenseNet-161	B-mode ultrasound	Accuracy: 92.0%	(19)
	GoogLeNet	Whole slide images	Accuracy: 99.3%	(38)
	Inception-v3	Vaginoscopic images	Accuracy: 90.4%	(75)
	MobileNet-v2	Clinical photographs	Accuracy: 77.8%	(48)
	RDP-Net	Histological images	Weighted F1-Score: 84.4%	(21)
	ResNet-18	Thoracic radiographs	AUC: 0.890	(43)
	YOLOv8	Blood smears	Macro F1-Score: 0.940	(63)
	Custom sequential	Skin clinical photographs	Accuracy: 98.4%	(67)
**Computational task**	**Architectural family**	**Input type**	**Reported performance**	**References**
	Custom sequential	Ocular clinical photographs	Accuracy: 84.4%	(61)
	YOLOv8-n	Renal ultrasound	Accuracy: 85.0%	(74)
	ResNet-50	Thoracic radiographs	Accuracy: 84.1%	(51)
	ResNet-50	Thoracic radiographs	Mean AUC: 0.820	(50)
	Custom CNN	Magnetic resonance scans	Sensitivity: 100.0%	(70)
	MSPPNet	iEEG signals	Sensitivity: 88.6%	(71)
Object detection	Faster R-CNN (ResNet-50)	Whole slide images	F1-Score: 0.840	(77)
	Faster R-CNN (ResNet-50)	Whole slide images	F1-Score: 0.750	(76)
	RetinaNet + ResNet-18	Whole slide images	F1-Score: 0.830	(37)
	Tiny-YOLOv4	Clinical photographs	mAP: 0.973	(78)
	YOLOv3	Blood smears	Concordance: 96.6%	(15)
	YOLOv5	Gait video frames	Accuracy: 97.0%	(24)
	1D-CNN	Thoraco-lumbar radiographs	Accuracy: 92.5%	(34)
Segmentation	Mask R-CNN	Thoracic radiographs	DSC: 97.4%	(46)
	U-Net Variant	Thoracic radiographs	DSC: 94.2%	(44)
	U-Net + ResNet34	Ocular clinical photographs	IoU: 76.0%/DSC: 86.0%	(59)
	U-Net + EfficientNet	Coxofemoral radiographs	DSC: 96.0%/IoU: 92.0%	(20)
	U-Net	Thoracic radiographs	IoU: 94.7%	(54)
	U-Net	Skin whole slide images	Dice: 88.0%	(39)
	U-Net/ResNet	Lymphoma whole slide images	Dice: 88.0%	(40)
Regression	SNN (ResNet34)	Wound clinical photographs	MAPE: 5.0% – 30.0%	(62)
	U-Net Variant	Thoracic radiographs	Mean bias: -0.09 vertebrae	(44)
	Keypoint Regressor	Coxofemoral radiographs	MAE: 0.0034 mm	(20)
	Mask R-CNN + Axes	Thoracic radiographs	PCC: 0.922/RMSE: 0.587	(46)
Multi-task/hybrid	HawksheadNet	Whole slide images	AUROC: 0.969	(40)
	ResNet-50	Whole slide images	F1-Score: 0.800	(35)
	Custom CNN/ResAttNet	CT scans	AUC: 0.939	(18)
	U-Net + EfficientNet	Whole slide images	Accuracy: 95.0%	(39)
	U-Net + Ensemble ML	Thoracic radiographs	IoU: 94.7%	(54)

When evaluating these performance findings globally, the exceptional efficacy peaks reported in binary tasks must be interpreted with caution. These elevated values are typically achieved under highly controlled experimental conditions or on heavily curated datasets, which may not reflect the natural variability of clinical practice. Furthermore, the notable performance drop when transitioning from binary problems to multi-class classification highlights a shared structural limitation: current models exhibit reduced precision when pathologies share similar visual features and minority classes lack sufficient training samples for robust algorithmic differentiation.

To provide a more meaningful assessment of the reliability of the reported performance, we explicitly analyzed the validation strategies employed across the 57 included studies. The overwhelming majority relied on internal validation: 54 studies utilized a standard hold-out (train-test) split, and 26 incorporated *k*-fold cross-validation. However, only six studies (10.5%) evaluated their models using external validation sets from independent institutions. Furthermore, among studies handling multiple images per animal, only seven explicitly reported utilizing patient-level splitting to prevent data leakage between training and testing sets. The scarcity of external validation and patient-level splitting suggests that the exceptionally high accuracy metrics frequently reported in the literature may be partially inflated due to dataset correlation. Consequently, while these models demonstrate strong proof-of-concept performance, their true generalizability and clinical reliability remain insufficiently tested.

A quantitative summary of the six-criterion risk-of-bias assessment across the 57 included studies showed that 55 studies (96.5%) obtained High Quality composite scores (9–12), while two studies (3.5%) obtained Moderate Quality scores (5–8). Scores across individual criteria were higher for model description and task-specific performance assessment than for external validation and data-partitioning procedures. For Q2, 48 studies (84.2%) received two points for model description, indicating that the architecture and main modeling procedures were described sufficiently. For Q4, 35 studies (61.4%) received two points for using task-specific performance metrics. In contrast, Q3 showed limited use of independent validation: only 6 studies (10.5%) evaluated model performance using independent out-of-distribution datasets, whereas 51 studies (89.5%) relied on internal resampling or hold-out validation. Q5 showed a similar limitation in data-partitioning procedures: only seven studies (12.3%) explicitly used patient-level data partitioning, while 50 studies (87.7%) did not report such partitioning. These findings indicate that high composite scores were mainly associated with clear model description and evaluation procedures. In contrast, independent validation and control of subject-level data dependence were reported less often.

### 3.4 Clinical and technological context of application

The vast majority of authors designed their algorithms with primary care in mind. In these settings, AI models were framed as decision-support tools for veterinary personnel rather than replacements for clinical evaluation. In Cardiology, they were primarily utilized for the initial detection of abnormalities such as left atrial enlargement (42), hypertrophic cardiomyopathy (47), and mitral valve disease (43). Applications were also identified for detecting pulmonary abnormalities on thoracic radiographs (50, 51) and infectious diseases such as coccidioidomycosis (53).

In Dermatology, clinical photographs were used to classify various types of skin lesions and support differential diagnosis (25, 67, 68). Ophthalmology also benefits from this approach, where automation enables the diagnosis of specific conditions such as retinal detachment (57), cataract classification (19), and the evaluation of epiphora and edema (59).

Another key role in primary care is the automated quantification of radiographic findings, which reduces manual clinical workload. This includes measuring the Vertebral Heart Size (VHS) for cardiomegaly assessment (44, 46) and supporting objective evaluation of joint dysplasia in both elbows (60) and hips (20). However, despite these diverse applications, the required infrastructure spans from mobile devices to dedicated cloud GPUs, and many studies omit specifying the hardware used, making it difficult to assess their true feasibility in resource-constrained clinics. From a geographical perspective, the overall scientific output of these developments is concentrated in high-income countries such as the United States, South Korea, and various European nations, as shown in Figure 5. The participation of emerging economies—including Turkey, India, Thailand, Brazil, and Algeria—is sparse, suggesting that the implementation of these tools heavily depends on installed computational infrastructure.

**Figure 5:**
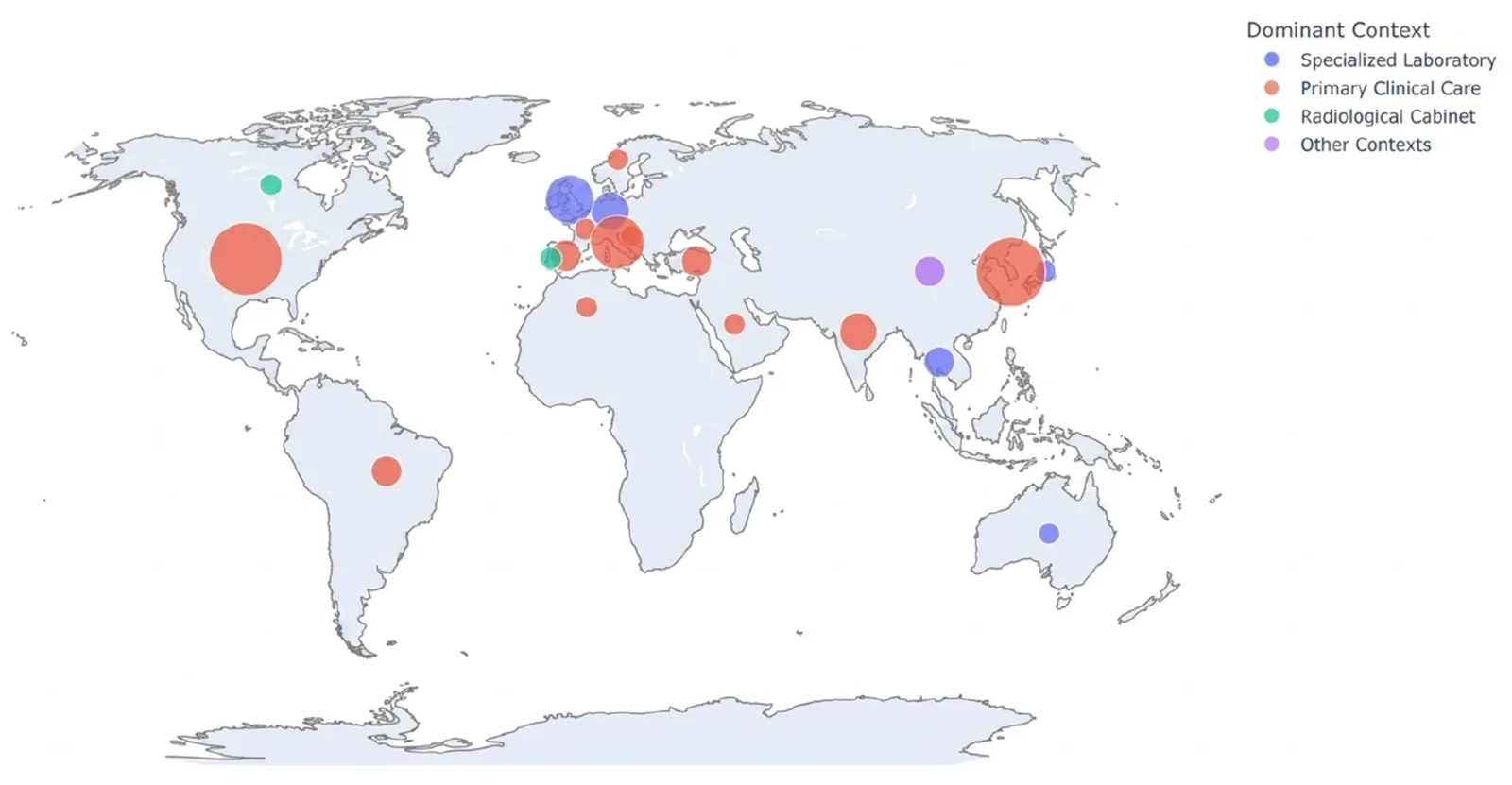
Geographical distribution and dominant clinical context by country.

The specialized laboratory was the second most frequent context among the reviewed studies. Applications were mainly concentrated in histopathology and cytology, where models were employed to segment, quantify, and classify tumor lesions (37, 39, 77). Studies aimed at detecting structures associated with neoplastic processes were also identified (40, 76). Differential diagnosis from microscopic preparations is also prominent, encompassing the typing of epithelial (23, 32) and mesenchymal (35, 38) neoplasms, as well as rigorous hematological analysis (63–65). Several of these studies reported using dedicated GPUs and high-performance computing (HPC) platforms, an infrastructure primarily associated with processing high-resolution histopathological images.

In the radiology suite, applications were identified for anatomical structure segmentation and computer-assisted image evaluation (18, 20, 54). In studies developed for emergency and triage settings, architectures such as DenseNet and YOLO were utilized to perform detection tasks with reduced processing times (24, 49, 55). However, not all studies provided sufficient information regarding inference times or the hardware used. Remote home monitoring proposals using wearable sensors were also found, where LSTM networks processed temporal data related to animal activity and behavior (72, 73).

Table 5 summarizes the identified application contexts, the roles assigned to the models, and the reported technological infrastructure.

**Table 5:** Clinical contexts, AI roles, and reported infrastructure.

Context	AI role	Technologies	Infrastructure	References
Primary clinical care	Quantification, diagnosis, screening, clinical support	DenseNet, EfficientNet, Inception, MobileNet, ResNet, Swin-T, U-Net, VGG, Xception, YOLO	CPU, edge/mobile, GPU, HPC/cloud, unstated	(16, 17, 19, 25, 42–48, 50–53, 56, 57, 59, 60, 62, 67–69, 74, 75, 78)
Specialized laboratory	Quantification, diagnosis, and prediction	CLAM, DenseNet, EfficientNet, Inception, MobileNet, ResNet, RetinaNet, U-Net, VGG, ViT, YOLO/R-CNN, 1D-CNN	GPU, HPC/cloud, unstated	(15, 22, 23, 32–41, 58, 63–66, 76, 77)
Radiology suite	Quantification, screening, clinical support	Custom CNN, ResAttNet, ResNet, U-Net	GPU, HPC/cloud, unstated	(18, 20, 54, 70)
Emergency and triage	Rapid diagnosis, screening	DenseNet, ResNet, VGG, YOLO	Edge/mobile, GPU, unstated	(24, 49, 55)
Remote monitoring	Continuous monitoring	1D-CNN, LSTM, ResNet	Edge/mobile, dedicated GPU, unstated	(61, 72, 73)
Other contexts	Seizure prediction	CNN-LSTM (MSPPNet)	Dedicated GPU	(71)

The infrastructure described across studies highlights a potential bottleneck for deploying some of these models in clinics with limited technological resources. Although several solutions were designed to support primary care, a portion of them relied on dedicated GPUs, cloud services, or high-performance computing platforms. Furthermore, numerous works failed to specify minimum hardware requirements, inference times, or execution costs. This lack of reporting hinders determining whether these models can run on conventional equipment. Consequently, future studies should evaluate model performance on resource-constrained devices and report the required technical specifications with greater precision.

## 4 Discussion

Collectively, the 57 selected studies demonstrate that the integration of deep learning into veterinary diagnostics depends on several interrelated factors: clinical data availability, architectural selection, performance evaluation methodologies, and technical deployment conditions. Broad reviews on artificial intelligence in veterinary medicine describe applications spanning clinical practice, biomedical research, and healthcare management (79, 80). Meanwhile, studies focused on diagnostic imaging indicate that much of the development has targeted the processing of radiographs, ultrasonography, clinical photographs, and microscopic imagery (81). Unlike these broad-scope reviews, the present study narrowed its focus to canine and feline patients, CNN and LSTM architectures, and diagnostic and clinical decision support. This targeted scope enabled us to directly correlate investigated pathologies with input data modalities, reported performance metrics, and technological infrastructure across studies. As summarized in Table 6, this review focuses on CNN and LSTM architectures applied to diagnostic and clinical decision support in dogs and cats. Restricting the scope to these two deep learning architectures allows comparison of their applications to spatial and temporal clinical data. In addition to model performance, this study examines computational deployment conditions, including reported hardware specifications, inference latency, and point-of-care use, to characterize the operational requirements described in the included studies. The analysis considers deployment and infrastructure characteristics alongside methodological quality.

**Table 6:** Structured comparison of the present review against recent related reviews on artificial intelligence in veterinary medicine.

Review	Methodological design	Included studies	Target species	Algorithmic scope	Clinical domain	Infrastructure evaluation
Akinsulie et al. (79)	Narrative review	—	All veterinary species	Broad AI and ML	Clinical practice, epidemiology, reproduction, and AMR	None
Akbarein et al. (80)	Narrative review	170	All small, large, and wildlife species	AI, ML, and DL models	Clinical, research, public health, and administration	Low with conceptual discussion only
Xiao et al. (31)	PRISMA systematic review	39	Canine, feline, bovine, and equine species	General DL including CNN, ANN, RNN, ViT, and LLM	General diagnostics across imaging, cytology, EHR, and sensors	Moderate with hardware mentioned in passing
Li et al. (82)	PRISMA-ScR scoping review	69	Companion animals as translational oncology models	AI, ML, and DL approaches	Veterinary oncology exclusively	Low to moderate focus on autoML accessibility
Burti et al. (26)	Narrative review	45	All species with focus on dogs and cats	AI and DL image architectures	Diagnostic imaging exclusively across radiology, ultrasound, CT, and MRI	Low focus on ethics and imaging workflows
Proposed review	PRISMA 2020 systematic review	57	Dogs and cats exclusively	CNN and LSTM deep learning architectures	Diagnostic and decision support across 11 clinical domains	High with systematic audit of hardware, latency, and deployment

The results reveal a heavy concentration of studies in Oncology, Cardiology, and Pulmonology. The prominence of oncological applications aligns with a recent review highlighting the rapid growth of models developed for tumor classification, mitotic figure detection, and histopathological image analysis (82). Similarly, reviews in clinical and digital pathology demonstrate that the ready availability of blood smears, cytological preparations, and digitized slides has fostered automated tool development (83, 84). In this review, eleven clinical domains were evaluated under unified criteria, revealing a higher representation of canine over feline subjects. However, this distribution reflects published research trends and does not imply that the most investigated pathologies are necessarily the most prevalent or prioritized in routine veterinary practice.

Architectural selection was intrinsically coupled with clinical data modality. CNNs were deployed across radiographs, ultrasonography, photographs, and microscopic images, whereas LSTMs and RNNs were applied exclusively to temporal signals. The widespread adoption of ResNet aligns with the use of residual connections to facilitate deep network training, though its prevalence does not establish it as the optimal architecture across all tasks (85). Conversely, LSTM networks are specifically designed to retain contextual information over long sequences, explaining their application in accelerometry, electroencephalography, and acoustic signals (86). Hybrid and multimodal frameworks were also identified. However, combining multiple data modalities introduces complex challenges related to data representation, alignment, and fusion (87). Compared to single-application reviews, this analysis enabled cross-modal comparison across seven clinical data modalities.

Accuracy, AUC, and F1-score metrics varied substantially based on computational task complexity and the specific validation protocols employed. Peak performance values were concentrated in binary classification problems, whereas multi-class tasks exhibited broader performance ranges. In imbalanced datasets, accuracy and F1-score can yield overly optimistic or incomplete insights; consequently, supplementing these metrics with indicators such as the Matthews Correlation Coefficient has been strongly advocated (88). Furthermore, clinical model evaluation should extend beyond discriminative power to incorporate calibration assessment and clinical utility analyses (89). External validation on out-of-distribution datasets and transparent reporting of evaluation procedures are essential prerequisites for transitioning experimental models into clinical practice (90). Heterogeneity in metrics, data partitioning, and task definitions across the reviewed literature precluded a meta-analytic quantitative comparison among models.

Our six-criterion risk-of-bias assessment identified limitations in the validation and data-partitioning procedures reported across the 57 included studies. Although 55 studies (96.5%) obtained High Quality composite scores, only six studies (10.5%) reported independent external validation, while 51 studies (89.5%) relied on internal resampling or hold-out validation. Patient-level data partitioning was explicitly reported in only seven studies (12.3%), whereas 50 studies (87.7%) did not report this procedure. These findings are particularly relevant for studies using multiple images, frames, or temporal samples from the same animal, because the absence of patient-level partitioning may allow samples from the same subject to occur across training and test sets, potentially resulting in optimistic estimates of model performance. Therefore, the high accuracy values reported in some classification studies, including values approaching 99%, should be interpreted in relation to the validation and data-partitioning procedures used. Future studies should place greater emphasis on independent external validation, patient-level data partitioning, and multi-center datasets to assess the generalizability of deep learning models under conditions that more closely reflect clinical practice.

Primary clinical care was the most frequently targeted deployment context, yet synthesized data do not support the conclusion that these models are immediately ready for point-of-care implementation. Several studies relied on dedicated GPUs, cloud computing, or high-performance computing platforms, whereas many omitted hardware requirements entirely. Veterinary radiology organizations emphasize that clinical AI integration demands rigorous validation, algorithmic transparency, continuous oversight, and active practitioner involvement in diagnostic decision-making (91). Establishing regulatory frameworks to monitor post-deployment model performance and prevent performance drift has also been identified as a priority (92). From a technological standpoint, localized processing via edge computing can reduce reliance on cloud infrastructure and minimize inference latency (93). However, edge deployment does not substitute for clinical validation. Therefore, clinical viability must be holistically evaluated alongside execution costs, inference latency, and hardware constraints.

This review has several limitations. The search query was restricted to Scopus and Web of Science, potentially omitting relevant literature indexed exclusively in other databases. Coverage disparities among academic search engines are well-documented in methodological research (94). Furthermore, inclusion was restricted to English-language, Open Access journal articles. While meta-epidemiological studies suggest that language restrictions often exert minimal bias on clinical review outcomes, this finding cannot be unconditionally generalized to regional veterinary literature (95). Additionally, restricting the timeframe to 2021–2026 and excluding classical machine learning techniques narrowed the technological scope of the synthesis. Finally, substantial heterogeneity in task formulations, performance metrics, target populations, sample sizes, and validation strategies precluded meta-analytic pooling, as quantitative synthesis requires a prior assessment of the degree and sources of heterogeneity (96).

## 5 Conclusions

This systematic review shows that artificial intelligence applications in veterinary diagnostic and clinical decision support are concentrated in specific clinical domains. Oncology accounted for 28.1% of the included studies, followed by cardiology with 15.8% and pulmonology with 12.3%. Image-based applications constituted a major component of the reviewed literature, with CNNs used across multiple data modalities and architectures such as ResNet applied in several of them. Binary classification tasks included the highest reported accuracy values, reaching up to 99%, whereas performance across multi-class tasks varied across studies. These differences were associated with variations in dataset characteristics, class distribution, task complexity, and evaluation protocols. The reported performance should therefore be interpreted in relation to the validation procedures and study settings. In particular, the limited use of external validation, prospective evaluation, standardized reporting of validation procedures, and real-world implementation restricts the assessment of model generalizability and clinical applicability. Further translation into clinical practice requires prospective multicenter studies, independent external validation, standardized reporting of inference performance and hardware requirements, and appropriate regulatory assessment before deployment. The reviewed studies were also concentrated in high-income countries, indicating differences in the geographic distribution of research activity. Broader application of artificial intelligence in veterinary medicine will therefore require not only further model evaluation but also greater access to open data resources and computational infrastructure in emerging economies.

## Figures and Tables

**Table:** 

Category	Abbreviation	Description
Deep neural networks	CNN	Convolutional Neural Network. Architectural family designed for processing spatial data, particularly images.
	LSTM	Long Short-Term Memory Network. Recurrent network variant specialized in processing temporal sequences.
	RNN	Recurrent Neural Network. Base architecture for sequential data, from which LSTM derives.
	ResNet	Residual Network. CNN family incorporating shortcut connections to mitigate the vanishing gradient problem in deep networks.
	DenseNet	Densely Connected Network. Variant in which each layer receives inputs from all preceding layers.
	EfficientNet	CNN family designed to simultaneously optimize network depth, width, and resolution, maximizing the trade-off between performance and computational cost.
	Inception	CNN architecture utilizing parallel modules with varying filter sizes to capture features across multiple scales.
	U-Net	CNN architecture with an encoder-decoder structure, widely used in medical image semantic segmentation tasks.
	VGG	Visual Geometry Group Convolutional Networks family. Characterized by sequential convolutional layers with small filters and substantial depth.
	YOLO	Real-Time Object Detector. Architecture that performs structure detection and localization in a single pass through the network.
	R-CNN	Region-based Convolutional Neural Network. Family of detectors that proposes candidate regions prior to classification.
	Transformer	Architecture based on attention mechanisms that processes data without recurrent structure, progressively adopted in computer vision.
Clinical input types	WSI	Whole Slide Image. High-resolution digitization of complete histopathological slides.
	H&E	Hematoxylin and Eosin histological stain, routinely used to visualize cellular morphology in microscopic preparations.
	RL	Right Lateral projection. Standard view in veterinary thoracic radiographs.
	LL	Left Lateral projection. Complementary view in thoracic radiographs.
	RNA-seq	RNA sequencing. Genomic technique that quantifies gene expression at a transcriptomic scale.
Evaluation metrics	Accuracy	Accuracy. Proportion of correct predictions over the total evaluated cases.
	AUC	Area Under the Curve. Indicator of overall discriminative capacity of a binary classifier.
	F1-Score	Harmonic mean between Precision and Sensitivity (Recall). Summarizes model performance when classes are imbalanced.
	Precision	Precision. Proportion of positive predictions that are truly positive.
	Recall/Sensitivity	Sensitivity/Recall. Proportion of actual positive cases correctly identified by the model.
	Specificity	Specificity. Proportion of actual negative cases correctly classified.
	mAP	mean Average Precision. Standard metric for evaluating object detectors.
	Dice/DSC	Dice Similarity Coefficient. Measures the overlap between predicted and reference segmentations.
	IoU	Intersection over Union. Metric quantifying the overlap between predicted and ground-truth masks.
	MAE	Mean Absolute Error. Regression metric averaging absolute differences between predicted and actual values.
	MAPE	Mean Absolute Percentage Error. Percentage version of MAE.
	MCC	Matthews Correlation Coefficient. Metric robust to class imbalance.
	Cohen's Kappa	Statistic measuring agreement between classifiers beyond chance, useful in multi-class contexts.
	AUROC	Area Under the Receiver Operating Characteristic Curve. Equivalent to AUC in multi-class contexts.
	FPR	False Positive Rate. Proportion of actual negative cases incorrectly classified as positive.
	NPV	Negative Predictive Value. Probability that a negative result is truly negative.

## Data Availability

The original contributions presented in the study are included in the article/Supplementary material, further inquiries can be directed to the corresponding author.
