# Supplementary material for: Deep learning for diagnostic and clinical decision support in companion animals: a systematic review of CNN- and LSTM-based approaches
Source: Front Vet Sci. 2026 Oct 22;13:1952589. doi: 10.3389/fvets.2026.1952589 (PMC13736258; doi:10.3389/fvets.2026.1952589)
Supplement: Supplementary file 1 [file Supplementary_file_1.pdf]

Supplementary Material

Reproducible search strategies

The complete search strategies used in Scopus and Web of Science are presented below. The search strings were adapted to the syntax of each database and were executed on April 23, 2026.

Scopus
(TITLE-ABS-KEY(Disease OR Pathology) AND TITLE-ABS-KEY(CNN OR "Convolutional Neural Network" OR LSTM OR "Long Short-Term Memory") AND TITLE-ABS-KEY(Prediction OR Detection OR Classification OR Diagnosis) AND TITLE-ABS-KEY(Dog OR Canine OR Cat OR Feline OR Veterinary)) AND PUBYEAR > 2020 AND PUBYEAR < 2027 AND (LIMIT-TO (DOCTYPE,"ar")) AND (LIMIT-TO (LANGUAGE,"English")) AND (LIMIT-TO (OA,"all"))

Web of Science
TS=(Disease OR Pathology) AND TS=(CNN OR "Convolutional Neural Network" OR LSTM OR "Long Short-Term Memory") AND TS=(Prediction OR Detection OR Classification OR Diagnosis) AND TS=(Dog OR Canine OR Cat OR Feline OR Veterinary) AND PY=(2021-2026) AND DT=Article AND LA=English AND OA=Open Access

Methodological quality assessment

The methodological quality and risk of bias assessment provided an overview of the strengths and limitations of the included studies. Table S1 presents the individual scores across the six assessment criteria, together with the resulting overall quality classification.

Table S1: Methodological quality and risk of bias assessment of the included studies

Study	Q1	Q2	Q3	Q4	Q5	Q6	Score	Quality
Al Mouiee et al. (2021)	2	2	1	2	2	1	10	High
Alanazi (2025)	2	2	1	2	1	1	9	High
Ancheta et al. (2025)	2	2	2	2	1	1	10	High
Banik et al. (2026)	2	2	2	2	1	1	10	High
Banzato et al. (2021a)	2	2	1	2	1	1	9	High
Banzato et al. (2021b)	2	2	1	2	1	1	9	High
Baruah and Boruah (2024)	2	1	1	2	1	1	8	Moderate
Bertram et al. (2022)	2	2	2	2	2	1	11	High
Biercher et al. (2021)	2	2	1	2	1	1	9	High
Buric et al. (2024)	2	1	1	2	1	1	8	Moderate
Burrai et al. (2023)	2	2	1	2	1	1	9	High
Cho (2025)	2	2	1	2	1	1	9	High
De Moraes Restani et al. (2025)	2	2	1	2	1	1	9	High
Dumortier et al. (2022)	2	2	1	2	1	1	9	High
Eddine Rahmoun et al. (2025)	2	2	1	2	1	1	9	High

Table S1 (continued)

Study	Q1	Q2	Q3	Q4	Q5	Q6	Score	Quality
Fragoso-Garcia et al. (2023)	2	2	1	2	1	1	9	High
Franco-Gonçalo et al. (2025)	2	2	1	2	1	1	9	High
Gao et al. (2023)	2	2	1	2	1	1	9	High
Hauback et al. (2025)	2	2	1	2	1	1	9	High
Hubbard-Perez et al. (2024)	2	2	1	2	1	1	9	High
Hwang et al. (2022)	2	2	1	2	1	1	9	High
Işık et al. (2023)	2	2	1	2	1	1	9	High
Istrate et al. (2025)	2	2	1	2	1	1	9	High
Jaworski et al. (2024)	2	2	1	2	1	1	9	High
K. Rajan et al. (2024)	2	2	1	2	1	1	9	High
Kovitvadhi et al. (2026)	2	2	2	2	1	1	10	High
Lee et al. (2025)	2	2	1	2	1	1	9	High
Liu et al. (2025)	2	2	1	2	1	1	9	High
Long et al. (2023)	2	2	2	2	1	1	10	High
Martinez et al. (2026)	2	2	1	2	1	1	9	High
Mekonnen et al. (2025)	2	2	1	2	2	1	10	High
Misaka et al. (2026)	2	2	1	2	1	1	9	High
Morisi et al. (2023)	2	2	1	2	2	1	10	High
Morissette et al. (2025)	2	2	1	2	1	1	9	High
Mozumder et al. (2024)	2	2	1	2	1	1	9	High
Müller et al. (2022)	2	2	1	2	1	1	9	High
Nam and Dong (2023)	2	2	1	2	1	1	9	High
Natarajan et al. (2025)	2	2	1	2	1	1	9	High
Okur et al. (2026)	2	2	1	2	1	1	9	High
Ott et al. (2021)	2	2	1	2	1	1	9	High
Park et al. (2024)	2	2	1	2	1	1	9	High
Park et al. (2025)	2	2	1	2	1	1	9	High
Pasupa et al. (2023)	2	2	1	2	1	1	9	High
Patkar et al. (2023)	2	2	1	2	1	1	9	High
Puget et al. (2025)	2	2	1	2	2	1	10	High
Rai et al. (2024)	2	2	1	2	1	1	9	High
Rho et al. (2023)	2	2	1	2	1	1	9	High
Smith et al. (2024)	2	2	1	2	1	1	9	High
Solomon et al. (2023)	2	2	2	2	2	1	11	High
Tahghighi et al. (2023)	2	2	1	2	1	1	9	High
Teague et al. (2023)	2	2	1	2	1	1	9	High
Unknown (2022)	2	2	1	2	1	1	9	High
Valente et al. (2023)	2	2	1	2	1	1	9	High
Valente et al. (2024)	2	2	1	2	1	1	9	High
Valente et al. (2026)	2	2	1	2	2	1	10	High
Wang et al. (2022)	2	2	1	2	1	1	9	High
Yu et al. (2024)	2	2	1	2	1	1	9	High

Note: Q1 = Data quality and representativeness; Q2 = Model and methodological description; Q3 = Validation strategy; Q4 = Appropriateness of performance or analytical metrics; Q5 = Bias and robustness control; Q6 = Real-world applicability. Each criterion was scored as 2 (adequately addressed), 1 (partially addressed), or 0 (inadequately addressed or not sufficiently reported). Overall methodological quality was classified as High (9–12), Moderate (5–8), or Low (0–4).

REFERENCES

Al Mouiee, D., Meijering, E., Kalloniatis, M., Nivison-Smith, L., Williams, R. A., Nayagam, D. A. X., et al. (2021). Classifying retinal degeneration in histological sections using deep learning. *Translational Vision Science & Technology* 10, 9

Alanazi, J. M. (2025). Detection of skin lesions in dogs using advanced convolutional neural network technology. *Indian Journal of Animal Research*

Ancheta, K., Psifidi, A., Yale, A. D., Le Calvez, S., and Williams, J. (2025). Deep-learning based morphological segmentation of canine diffuse large b-cell lymphoma. *Frontiers in Veterinary Science* 12, 1656976

Banik, M., Kreutz-Delgado, K., Mohanty, I., Brown, J. B., and Singh, N. (2026). Adaptive example selection for prototype based explainable mitosis detection in digital pathology. *Scientific Reports* 16, 9481

Banzato, T., Wodzinski, M., Burti, S., Osti, V. L., Rossoni, V., Atzori, M., et al. (2021a). Automatic classification of canine thoracic radiographs using deep learning. *Scientific Reports* 11, 3964

Banzato, T., Wodzinski, M., Tauceri, F., Donà, C., Scavazza, F., Müller, H., et al. (2021b). An ai-based algorithm for the automatic classification of thoracic radiographs in cats. *Frontiers in Veterinary Science* 8, 731936

Baruah, D. K. and Boruah, K. (2024). Early detection of canine babesia from red blood cell images using deep ensemble learning. *Journal of Electronics, Electromedical Engineering, and Medical Informatics* 6, 509–523

Bertram, C. A., Aubreville, M., Donovan, T. A., Bartel, A., Wilm, F., Marzahl, C., et al. (2022). Computer-assisted mitotic count using a deep learning–based algorithm improves interobserver reproducibility and accuracy. *Veterinary Pathology* 59, 211–226

Biercher, A., Meller, S., Wendt, J., Caspari, N., Schmidt-Mosig, J., De Decker, S., et al. (2021). Using deep learning to detect spinal cord diseases on thoracolumbar magnetic resonance images of dogs. *Frontiers in Veterinary Science* 8, 721167

Buric, M., Grozdanic, S., and Ivasic-Kos, M. (2024). Diagnosis of ophthalmologic diseases in canines based on images using neural networks for image segmentation. *Heliyon* 10, e38287

Burrai, G. P., Gabrieli, A., Polinas, M., Murgia, C., Becchere, M. P., Demontis, P., et al. (2023). Canine mammary tumor histopathological image classification via computer-aided pathology. *Animals* 13, 1563

Cho, O. H. (2025). Sequential convolutional neural network techniques to improve detection of lesions in dogs. *Indian Journal of Animal Research* 59

De Moraes Restani, G., Tavares De Brito Nery Jaworski, L., Rodrigues Froes, T., and Ferrari De Oliveira, L. (2025). Classification of left atrial enlargement in canine radiographs using artificial intelligence. *IEEE Access* 13, 191609–191618

Dumortier, L., Guépin, F., Delignette-Muller, M.-L., Boulocher, C., and Grenier, T. (2022). Deep learning in veterinary medicine, an approach based on cnn to detect pulmonary abnormalities from lateral thoracic radiographs in cats. *Scientific Reports* 12, 11418

Eddine Rahmoun, D., Abdel Maksoud, M. K. M., Lieshchova, M., and Mylostyvyi, R. (2025). Applications of artificial intelligence in veterinary anatomical pathology. *Acta Veterinaria Eurasia* 51, 1–7

Fragoso-Garcia, M., Wilm, F., Bertram, C. A., Merz, S., Schmidt, A., Donovan, T., et al. (2023). Automated diagnosis of 7 canine skin tumors using machine learning on h&e-stained whole slide images. *Veterinary Pathology* 60, 865–875

Franco-Gonçalo, P., Leite, P., Alves-Pimenta, S., Colaço, B., Gonçalves, L., Filipe, V., et al. (2025). A computer-aided approach to canine hip dysplasia assessment: Measuring femoral head–acetabulum distance with deep learning. *Applied Sciences* 15, 5087

Gao, Y., Liu, A., Wang, L., Qian, R., and Chen, X. (2023). A self-interpretable deep learning model for seizure prediction using a multi-scale prototypical part network. *IEEE Transactions on Neural Systems and Rehabilitation Engineering* 31, 1847–1856

Hauback, M. N., Huynh, B. N., Steiro, S. E. D., Groendahl, A. R., Bredal, W., Tomic, O., et al. (2025). Deep learning can detect elbow disease in dogs screened for elbow dysplasia. *Veterinary Radiology & Ultrasound* 66, e13465

Hubbard-Perez, M., Luchian, A., Milford, C., and Ressel, L. (2024). Use of deep learning for the classification of hyperplastic lymph node and common subtypes of canine lymphomas: A preliminary study. *Frontiers in Veterinary Science* 10, 1309877

Hwang, S., Shin, H. K., Park, J. M., Kwon, B., and Kang, M.-G. (2022). Classification of dog skin diseases using deep learning with images captured from multispectral imaging device. *Molecular & Cellular Toxicology* 18, 299–309

Işık, A. H., Özmen, Ö., Eskicioğlu, Ö. C., Işık, N., and Melenli, S. (2023). Classification and diagnosis of mammary tumors in dogs using deep learning techniques. *Traitement du Signal* 40, 1747–1754

Istrate, A., Constantinescu, R., Anandavel, L., Rajeshkumar Tandel, S., Dye, S., and Dye, C. (2025). A novel machine learning model for the automated diagnosis of nasal pathology in canine patients. *Animals* 15, 1718

Jaworski, L. T. d. B. N., Restani, G. d. M., de Oliveira, L. F., and Froes, T. R. (2024). Evaluation of the effectiveness of two neural networks (resnet50v2 and inception v3) in identifying left atrial enlargement in adult dogs. *Archives of Veterinary Science* 29, 1–8

K. Rajan, B., G., V., Harshan M., H., and Swaminathan, R. (2024). Augmenting interpretation of vaginoscopy observations in cycling bitches with deep learning model. *BMC Veterinary Research* 20, 401

Kovitvadhi, A., Chawaphanth, Y., Boonnark, P., Wongsuppabut, K., Siripattarapravat, K., and Thainimit, S. (2026). Deep learning under severe class imbalance. *Computer Methods and Programs in Biomedicine Update* 9, 100248

Lee, S.-Y., Lee, S., Kim, S.-H., Chang, H., Cho, W.-Y., Ryu, M.-O., et al. (2025). Deep learning-based evaluation of the severity of mitral regurgitation in canine myxomatous mitral valve disease patients using digital stethoscope recordings. *BMC Veterinary Research* 21, 326

Liu, C., Cai, L.-H., Shen, Y.-F., Li, Z., He, Z.-J., Chen, X.-Y., et al. (2025). Deep learning-based gait recognition and evaluation of the wounded. *Disaster Medicine and Public Health Preparedness* 19, e272

Long, J., Ranganathan Ganakammal, S., Jones, S. E., Kothandaraman, H., Dhawan, D., Ogas, J., et al. (2023). ctulip: application of a human-based rna-seq primary tumor classification tool for cross-species primary tumor classification in canine. *Frontiers in Oncology* 13, 1216892

Martinez, R., Amezcua, K. L., Hernandez Torres, S. I., Winter, T., Yankin, I., Venn, E., et al. (2026). Artificial intelligence models for point-of-care ultrasound diagnostics in dogs. *Frontiers in Veterinary Science* 13, 1729114

Mekonnen, H. T., Puig, N., Elson, A., López, E., Martínez, P., Campos, S., et al. (2025). Deep learning framework for vertebral heart size and cardiothoracic ratio estimation in dogs and cats using thoracic radiographs. *Frontiers in Veterinary Science* 12, 1612338

Misaka, R., Yoshida, T., Tagawa, M., Iwasaki, R., Komatsu, Y., and Kayano, M. (2026). Deep learning models for image classification of lymphoma: a pilot study in canine. *Journal of Veterinary Medical Science* 88, 314–321

Morisi, A., Rai, T., Bacon, N. J., Thomas, S. A., Bober, M., Wells, K., et al. (2023). Detection of necrosis in digitised whole-slide images for better grading of canine soft-tissue sarcomas using machine-learning. *Veterinary Sciences* 10, 45

Morissette, E., Penn, C. D., Sedlak, R. A. H., Rhodes, A. J., Tippetts, D. S., Loenser, M., et al. (2025). Point-of-care platform integrated with deep-learning, convolutional neural network algorithms effectively evaluates canine and feline peripheral blood smears. *American Journal of Veterinary Research* 86

Mozumder, M. A. I., Theodore Armand, T. P., Sumon, R. I., Imtiyaj Uddin, S. M., and Kim, H.-C. (2024). Automated pipeline for robust cat activity detection based on deep learning and wearable sensor data. *Sensors* 24, 7436

Müller, T. R., Solano, M., and Tsunemi, M. H. (2022). Accuracy of artificial intelligence software for the detection of confirmed pleural effusion in thoracic radiographs in dogs. *Veterinary Radiology & Ultrasound* 63, 573–579

Nam, M. G. and Dong, S.-Y. (2023). Classification of companion animals' ocular diseases. *IEEE Access* 11, 143948–143955

Natarajan, S. S., Guruvayurappan, G., Jeyaselvarayan, J., Lakshmikanth, G., Danciulescu, D., Stoian, G., et al. (2025). Real time disease detection for cattles and pets and tool for veterinary assistance and farmers. *Traitement du Signal* 42, 1421–1435

Okur, S., Baykal, B., Akçora, Y., Modoğlu, E., Kibar, B., İlgün, M., et al. (2026). A comparative analysis of deep convolutional networks for automated diagnosis of retinal detachment in dogs. *Veterinary Ophthalmology* 29, e70176

Ott, J., Bruyette, D., Arbuckle, C., Balsz, D., Hecht, S., Shubitz, L., et al. (2021). Detecting pulmonary coccidioidomycosis with deep convolutional neural networks. *Machine Learning with Applications* 5, 100040

Park, J., Cho, H., Ji, Y., Lee, K., and Yoon, H. (2024). Development of a deep learning model for automatic detection of narrowed intervertebral disc space sites in caudal thoracic and lumbar lateral x-ray images of dogs. *Frontiers in Veterinary Science* 11, 1453765

Park, S., Go, S., Kim, S., and Shim, J. (2025). Deep learning-based classification of canine cataracts from ocular b-mode ultrasound images. *Animals* 15, 1327

Pasupa, K., Vatathanavaro, S., and Tungjitnob, S. (2023). Convolutional neural networks based focal loss for class imbalance problem: A case study of canine red blood cells morphology classification. *Journal of Ambient Intelligence and Humanized Computing* 14, 15259–15275

Patkar, S., Beck, J., Harmon, S., Mazcko, C., Turkbey, B., Choyke, P., et al. (2023). Deep domain adversarial learning for species-agnostic classification of histologic subtypes of osteosarcoma. *The American Journal of Pathology* 193, 60–72

Puget, C., Ganz, J., Ostermaier, J., Conrad, T., Parlak, E., Bertram, C. A., et al. (2025). Artificial intelligence can be trained to predict c-kit-11 mutational status of canine mast cell tumors from hematoxylin and eosin-stained histological slides. *Veterinary Pathology* 62, 152–160

Rai, T., Morisi, A., Bacci, B., Bacon, N. J., Dark, M. J., Aboellail, T., et al. (2024). Keeping pathologists in the loop and an adaptive f1-score threshold method for mitosis detection in canine perivascular wall tumours. *Cancers* 16, 644

Rho, J., Shin, S.-M., Jhang, K., Lee, G., Song, K.-H., Shin, H., et al. (2023). Deep learning-based diagnosis of feline hypertrophic cardiomyopathy. *PLOS ONE* 18, e0280438

Smith, A., Carroll, P. W., Aravamuthan, S., Walleser, E., Lin, H., Anklam, K., et al. (2024). Computer vision model for the detection of canine pododermatitis and neoplasia of the paw. *Veterinary Dermatology* 35, 138–147

Solomon, J., Bender, S., Durgempudi, P., Robar, C., Cocchiaro, M., Turner, S., et al. (2023). Diagnostic validation of vertebral heart score machine learning algorithm for canine lateral chest radiographs. *Journal of Small Animal Practice* 64, 769–775

Tahghighi, P., Norena, N., Ukwatta, E., Appleby, R. B., and Komeili, A. (2023). Automatic classification of symmetry of hemithoraces in canine and feline radiographs. *Journal of Medical Imaging* 10

Teague, J., Socia, D., An, G., Badylak, S., Johnson, S., Jiang, P., et al. (2023). Artificial intelligence optical biopsy for evaluating the functional state of wounds. *Journal of Surgical Research* 291, 683–690

Unknown (2022). Development of an artificial neural network for the detection of supporting hindlimb lameness: A pilot study in working dogs. *Animals* 12, 1755

Valente, C., Wodzinski, M., Guglielmini, C., Poser, H., Chiavegato, D., Zotti, A., et al. (2023). Development of an artificial intelligence-based method for the diagnosis of the severity of myxomatous mitral valve disease from canine chest radiographs. *Frontiers in Veterinary Science* 10, 1227009

Valente, C., Wodzinski, M., Guglielmini, C., Poser, H., Chiavegato, D., Zotti, A., et al. (2024). Development of an artificial intelligence-based algorithm for predicting the severity of myxomatous mitral valve disease from thoracic radiographs by using two grading systems. *Research in Veterinary Science* 178, 105377

Valente, C., Wodzinski, M., Guglielmini, C., Poser, H., Zotti, A., Mastromattei, N., et al. (2026). Development of an artificial intelligence-based algorithm for the detection of left atrial enlargement from feline thoracic radiographs. *Veterinary Quarterly* 46, 2611847

Wang, H., Atif, O., Tian, J., Lee, J., Park, D., and Chung, Y. (2022). Multi-level hierarchical complex behavior monitoring system for dog psychological separation anxiety symptoms. *Sensors* 22, 1556

Yu, H., Lee, I.-G., Oh, J.-Y., Kim, J., Jeong, J.-H., and Eom, K. (2024). Deep learning-based ultrasonographic classification of canine chronic kidney disease. *Frontiers in Veterinary Science* 11, 1443234
